# Altered Cingulum Functioning in Major Depressive Disorder Patient With Suicide Attempts: A Resting-State Functional Magnetic Resonance Imaging Study

**DOI:** 10.3389/fnins.2022.849158

**Published:** 2022-03-28

**Authors:** Chunxia Yang, Yajuan Duan, Lei Lei, Penghong Liu, Aixia Zhang, Gaizhi Li, Ning Sun, Yikun Wang, Zhifen Liu, Kerang Zhang

**Affiliations:** ^1^Department of Psychiatry, First Hospital of Shanxi Medical University, Taiyuan, China; ^2^The First Hospital, Shanxi Medical University, Taiyuan, China; ^3^Nursing College of Shanxi Medical University, Taiyuan, China

**Keywords:** major depressive disorder, resting-state fMRI, regional homogeneity (ReHo), suicidal attempts, cingulum functioning

## Abstract

**Background:**

Major depressive disorder (MDD) with suicide attempts (SA) poses a significant public health issue. This study aims to identify neurobiological markers for MDD with SA on resting-state brain functional magnetic resonance imaging (rs-fMRI).

**Methods:**

Fifty-one unmedicated adult MDD participants, 27 with SA on the Beck Scale for Suicidal Ideation and 24 without SA, underwent rs-fMRI scanning. A group of 30 healthy controls (HC) matched for age, gender, and education-level with MDD were chosen. A whole brain analysis of regional homogeneity (ReHo) was performed on subjects to identify regions where brain activity was associated with SA. Multiple comparison analysis was performed for ReHo. Pearson’s correlation analysis was performed between HAMD-SA scores and ReHo. The statistical significance level was set at *p* < 0.05.

**Results:**

We examined whether there were significant differences among the three groups in whole brain ReHo during resting state. Subjects with SA showed significant increase of ReHo in the right Cingulum Post in comparison with those without SA. Subjects with SA showed significant decrease of ReHo in the right Cingulate Gyrus/Precuneus in comparison with HC. The mean ReHo from the significant brain region was associated with HAMD-SA (item 3 of the HAMD) scores (*r* = 0.349, *P* = 0.012) but was not associated with HAMD-24 scores.

**Conclusion:**

These results indicate that SA is associated with altered resting-state brain activity. The pattern of elevated activity in the cingulum functioning may be related to SA. Identifying cingulum activity associated with SA may help to elucidate its pathogenesis and etiology.

## Introduction

Major depressive disorder (MDD) is a worldwide widespread psychiatric disorder associated with premature death by suicide (Saraceno, 2002; Kalin, 2020). The lifetime risk of suicide in patients with MDD ranges from 5 to 11% (Angst et al., 2005; Isometsä, 2014). Approximately 40–70% of those who have attempted or committed suicide were diagnosed with major depressive disorder (Rihmer, 2007). Recent studies suggest that the lifetime prevalence of suicide attempts (SA) in MDD was as high as 31% worldwide (Dong et al., 2019). Although the social and personal costs of suicidal behavior are devastating, clinically, suicide risk in MDD patients is predicted based on a few limited scale tests. However, these tests are largely dependent on the subjective wishes of the patients. Also, patients may be inclined to avoid discussing SA with clinicians (Zalsman et al., 2016). Therefore, it is important to identify markers associated with SA, which may help to develop tests to assess the risk of suicide and also have the potential to create more targeted therapeutic strategies to reverse SA (Schmaal et al., 2020).

In an attempt to identify factors contributing to suicidal behavior, an increasing number of researchers have studied neurobiological markers pointing to functional and structural alterations in the limbic zone of MDD patients with suicidal behavior (Cao et al., 2016; Zhang et al., 2016). Meanwhile, previous studies also found that both psychotherapy (Chaïb et al., 2020) and psychoactive medication (Zalsman et al., 2016) could reduce the risk of suicide. However, there are no objective indicators to quantify this risk yet. Therefore, elucidating the neural basis of suicidal behavior in MDD may provide insights into early intervention and treatment. Resting state functional magnetic resonance imaging (rs-fMRI) studies have found that SA often occurs during mind rest phases, brain processes that occur when subjects are not engaged in any specific mental task (Rush and Beck, 1978). Previous studies showed that SA was associated with a pattern of low self-esteem (Cox et al., 2004; Bhar et al., 2008). Another study showed that two dimensions of rumination, brooding, and reflection, were predictors of suicidal ideation (Miranda and Nolen-Hoeksema, 2007). Moreover, rs-fMRI study explained the neural substrates of depressive rumination and explicit account of functional abnormalities in sgPFC in MDD (Hamilton et al., 2015). Therefore, rs-fMRI is particularly beneficial in finding SA in MDD related brain regions.

In magnetic resonance imaging (MRI), a powerful tool to explore alterations in neural circuits is regional homogeneity (ReHo), which reflects statistical similarity in spontaneous neural activity between spatially adjacent brain tissues (Zhu et al., 2005). ReHo is believed to reflect anatomical, morphological, and intrinsic geometric similarities and topological functional interactions of local brain structures. Abnormal ReHo reflects changes in the temporal aspects of regional neural activity (Jiang and Zuo, 2016). At present, the studies of brain function mainly focus on evaluating local functional changes, and ReHo is used as a measure of regional synchronization of the fMRI time course, and has been widely used in many studies on MDD. ReHo alterations in the prefrontal cortex, thalamus, right supplementary motor area, and primary visual, auditory, and motor cortices have been detected in MDD. A recent study found that lower ReHo in the postcentral gyrus was associated with depressive symptoms in MDD. In addition, a recent study (Xia et al., 2019) provided some evidence for differentiating subgroups of MDD. ReHo may be a transdiagnostic neurobiological basis for reproducible alterations in the assessment of underlying depressive symptoms.

Research has focused on investigating dynamic functional connectivity or networks, which can provide information about dynamic tissue changes in brain strength or space (Bassett and Sporns, 2017). Studies in depressed patients have linked SA to impulsive behavior and executive and emotional processing dysfunction (Myung et al., 2016; Johnston et al., 2017). Notably, executive functions and emotional processing involve brain regions such as the orbitofrontal cortex, anterior cingulate cortex, dorsolateral prefrontal cortex and temporal polar gyrus (Rogers et al., 2004; Olson et al., 2007). Frontal limbic (Du et al., 2017) and orbitofrontal thalamic functional connectivity (Kim et al., 2017) and frontal cortical white matter connectivity (Myung et al., 2016) were reduced in patients with SA compared to MDD patients without SA. Convergent findings suggest the presence of structural and fMRI abnormalities in MDD SA patients (Myung et al., 2016; Du et al., 2017; Kim et al., 2017). The reason for the inconsistency of these research results may be due to the limited research indicators or the different focus of the research objects, some teenagers (Ordaz et al., 2018) and women (Wei et al., 2018). However, there are a few promising studies (Cao et al., 2015; Chen et al., 2021) on suicide and ReHo indicators, which in turn encourages additional related research.

In an effort to characterize MDD patients with concomitant SA, we applied ReHo on resting state fMRI of MDD patients with and without SA. We sought to determine (1) whether MDD patients with SA show a different pattern of local consistency than MDD patients without SA and (2) whether the altered ReHo values could provide a neural marker to predict the severity of SA. By studying ReHo features in MDD patients with SA, we expect to delineate brain regions associated with SA that have the potential to be targeted for subsequent therapies. We also hope to shed further light on the biological details of the brains of MDD patients with SA.

## Materials and Methods

### Participants

The participants consisted of 51 first-episode, drug-naive patients with MDD. All of these patients were recruited from the Department of Psychiatry in the First Hospital of Shanxi Medical University between December 2018 and July 2019. Independent diagnoses by at least two consultant psychiatrists according to Diagnostic and Statistical Manual of Mental Disorders Fourth Edition (DSM-IV) criteria for MDD. The patients were also assessed with the Chinese Version of the Modified Structured Clinical Interview for DSM-IV TR Axis I Disorders Patient Edition (SCID-I/P, 11/2002 revision). At the same time, the subjects were interviewed using the 24-item HAMD. All of the subjects included in this study meet the following inclusion criteria: (1) aged from 18 to 65 years old; (2) right-handed; (3) diagnosed with first-episode, drug-naive patients based on the DSM-IV criteria; (4) HAMD-17 score >17 and HAMA-14 score <14. The exclusion criteria were: (1) meeting DSM-IV axis I psychiatric disorders; (2) with severe organic diseases such as neurological diseases; (3) obvious impulsivity, or uncooperativeness; (4) pregnant women; (5) contraindications for MRI scans.

The patients were divided into two groups based on whether or not they had a history of suicide attempts. Suicide attempt is defined as a self-destructive act leading to physical harm with some degree of intention to die. Accordingly, 24 patients who attempted suicide were categorized in the group. On the other hand, 27 patients who never attempted suicide were classified in the non-SA group. The Scale for Suicidal Ideation (SSI) was used to assess suicidal ideation as well as the risk of suicide which was not required for the non-SA and HCs group.

None of the subjects were excluded due to excessive head motion during the fMRI scan. In order to explore neurobiological markers for MDD with SA on rs-fMRI, 30 age-, gender-, and education-level-matched healthy controls were selected. Excluded subjects were those who were left-handed, had mental disorders, a neurological illness, or showed abnormalities on brain images.

Written informed consent was obtained from each participant and consent from each participant’s guardian was also obtained prior to data acquisition. The Ethical Committee for Medicine of the First Hospital of Shanxi Medical University approved this study.

### Magnetic Resonance Imaging Data Acquisition

Data preprocessing was conducted using an A MAGNETOM Trio Tim 3.0 T (Siemens Medical Solutions, Germany) with a 12-channel birdcage head coil located. The head of participants was positioned within a 32-channel head coil. A 3DFLASH sequence was used to obtain high resolution trasaxial T1-weighted anatomical images for voxel-based morphometry (VBM) with the following parameters: 120 sagittal slices, TR = 14 ms, TE = 4.92ms, thickness/skip = 1.5/0.3 mm, FOV = 230 mm × 230 mm, matrix = 256 × 192 mm, flip angle = 25°. The rs-fMRI was performed using an echo planar imaging (EPI) sequence with the following parameters: TR = 2,000 ms, TE = 30 ms, Flip angle = 70°, FOV = 24 cm × 24 cm, matrix = 64 × 64, section thickness = 3 mm, slice gap = 2 mm, acquired over 6 min and 212 volumes were obtained. Anatomic images were obtained with 3D MPRAGE sequence for co-registration with the functional data. The fMRI images were pre-processed in SPM5 (statistics parameter mapping^1^) and REST software for motion correction, band-pass filter (0.01–0.1 Hz), image normalization and 4 mm Gaussian spatial smoothing after Reho calculation. During the scan, all subjects were confirmed that they did not fall asleep.

### Regional Homogeneity Analysis

Regional homogeneity is based on the concept that BOLD signal fluctuations in a particular region reflect activity close to neurons at the same frequency, and this time synchronization is limited to groups of neurons performing related functions (Zang et al., 2004). We used the DPARSF software to calculate the ReHo. Individual ReHo maps were generated by calculating Kendall’s coefficient of concordance (KCC) of the time series of a given voxel with those of its nearest neighbors (26 voxels) in a voxel-wise analysis. Assuming that a voxel is similar to its neighbors in time, the consistency and similarity of each individual is assessed by calculating the KCC of the time series between a given voxel and its neighbors in voxel analysis. After the ReHo map was calculated on the basis of voxel-by-voxel, the standardized ReHo images were then spatially smoothed with a Gaussian kernel of 8 × 8 × 8 mm^3^ full width at half-maximum. Finally, low-frequency fluctuations (LFFs) within a functional cluster were synchronized with neighboring voxels.

### Statistical Analyses

All statistical analyses were performed using IBM SPSS Statistics Version 23.0 (SPSS23.0). One-way ANOVAs were conducted to detect the differences among the three groups in terms of age and, years of education. An X^2^-test was used to estimate group differences in gender. *T*-tests were conducted to compare the total HAMD score between the two patient groups. A multiple comparison analysis was performed to analyze the ReHo. The ReHo between the patient groups and controls were examined using one-way ANOVA analysis followed by *post-hoc* two-sample *t*-tests. The statistical significance level was set at *p* < 0.05.

To assess the effect of independent of SA, the value of the suicide item (item 3) of the HAMD was subtracted from the HAMD score in order to yield a clinical variable (HAMD-SA) for further analyses. Pearson’s correlation analysis was performed between HAMD-SA scores and ReHo.

## Results

We examined whether there were differences among the three groups in whole brain ReHo during a resting state. Subjects with SA showed a significant increase of in ReHo in the right Cingulum Post compared to those without SA. Subjects with SA showed a significant decrease of in ReHo in the right Cingulate Gyrus/Precuneus compared to HC. The mean ReHo from the significant brain region was associated with HAMD-SA scores (*r* = 0.349, *P* = 0.012) but was not associated with HAMD-24 scores.

### Demographic Data Comparisons

The MDD patients and HC were comparable in age, gender, and years of education with no significant differences. There were no significant differences between the patients with SA and without SA in their total HAMD-24 scores. The average SSI scores of with Suicide Attempts group was 10.04 ± 1.78 (Table 1).

**TABLE 1:** Demographic and clinical characteristics of all participants.

Variable	With suicide attempts group (*n* = 24)	Without suicide attempts symptoms group (*n* = 27)	HCs group (*n* = 30)	x^2^/t/F-Value	*P*-value
Gender (M/F)	8/16	14/13	16/14	1.26	0.289^a^
Age (years)	33.46 ± 9.47	30.96 ± 11.68	32.83 ± 8.42	0.45	0.640^b^
Education (years)	13.21 ± 4.45	13.41 ± 4.72	15.63 ± 3.41	2.90	0.061^b^
HAMD-17 total scores	20.83 ± 3.61	23.00 ± 3.09	−	1.06	0.308^c^
HAMD-24 total scores	26.54 ± 4.21	27.93 ± 3.97	−	0.14	0.714^c^

*^a^P-value for chi-square test.*

*^b^P-values for one-way ANOVA.*

*^c^P-values for two-sample t-test.*

### Regional Homogeneity Regions Differences in Suicide Attempts Symptoms Group, Non-suicide Attempts Symptoms Group, and Healthy Controls Group

**FIGURE 1:**
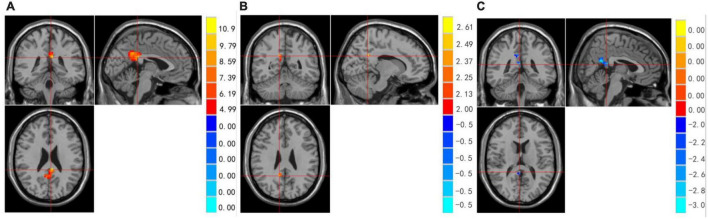
**(A–C)** Regions showing significant differences in ReHo among MDD with/without SA and healthy controls. Red represents HCs > with suicide attempts group brain area, blue represents without suicide attempts group < with suicide attempts group brain area.

We examined whether there were differences among the three groups in whole brain ReHo during resting state. Significant differences in ReHo were observed among the three groups for the right Cingulum Post (Table 2 and Figure 1A).

**TABLE 2:** Regions showing significant differences in regional homogeneity (ReHo) among major depressive disorder (MDD) with/without suicide attempts (SA) and healthy controls.

Area		Cluster size (voxels) mm^3^	BA	Side	MNI Co-ordinates^a^			F/T-value^b^
					x	y	z	
**Differences among three groups**								
HCs > with suicide attempts group	Cingulum post	4,617	23	Left	−3	−39	24	11.6288
	Cingulate Gyrus/Precuneus	189	−	Right	12	−51	27	2.7282
Without suicide attempts group < with suicide attempts group	Cingulum Post	1,188	−	Right	3	−42	15	−3.2385

*^a^Co-ordinates of primary peak locations in the Montreal Neurological Institute space.*

*^b^T-statistical value of peak voxel showing ReHo differences between groups.*

*BA, Brodmann area.*

*H represents healthy controls, Y represents depressed patients with SA, and N represents depressed patients without SA.*

Compared to the non-SA group, the SA group showed increased brain activity in the Right Cingulum Post (see Table 2 and Figure 1B). Compared to the HCs group, the SA group showed decreased brain activity in the right Cingulate Gyrus/Precuneus (see Table 2 and Figure 1C).

### Correlations Between HAMD-SA (item 3 of the HAMD) Scores and Regional Homogeneity

**FIGURE 2:**
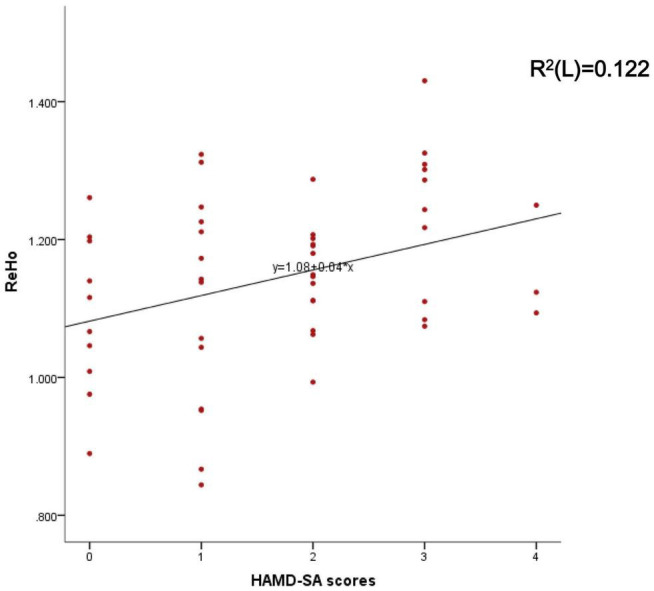
Represents the correlation between the mean ReHo of the right Cingulate Gyrus and HAMD-SA scores.

Mean ReHo from the significant brain region was associated with HAMD-SA (item 3 of the HAMD) scores (*r* = 0.349, *P* = 0.012) (see Figure 2) but was not associated with HAMD-24 scores.

## Discussion

At the initial assessment, because patients and clinicians may be relatively unaware of each other, suicide patients may deny SA during an interview. Some patients may deliberately obstruct interventions to prevent suicide (Brook et al., 2006). Identifying neurophysiologic markers is of great importance for objectively diagnosing SA in MDD patients. The results of our present study show that the SA group demonstrated increased local consistency of neural activity in the right cingulate relative to the NSA group; however, it demonstrated less local consistency in the right Cingulate/Precuneus relative to the HC group. In addition, the mean ReHo of right cingulate from the significant brain region correlated with the HAMD-SA score, but was not associated with HAMD-24 scores. Finding these ReHo-altered functional brain regions may shed light on the pathophysiological mechanism of suicide in MDD patients.

Cingulate has been the subject of intense research, with anterior cingulate cortex (ACC) volume loss being one of the most consistent findings (Arnone et al., 2012). ACC is involved in cognitive functioning and is executive functioning (Breukelaar et al., 2017), and impaired executive functioning may be a neuropsychological risk factor for suicidal behavior (Westheide et al., 2008). It has been reported that there was a volume decrease in the rostral anterior cingulate in patients with suicidal MDD compared to non-suicidal MDD patients (Wagner et al., 2011; Li et al., 2019). An autopsy study revealed that the number and length of dendritic branches in the anterior cingulate gyrus were significantly reduced in depressed suicide completers compared to controls (Hercher et al., 2010). A recent study reported decreased ACC activity in adolescents with a history of suicide attempts and depression compared to adolescents with a history of depression only (Pan et al., 2011). However, our study illustrates increasing right cingulate functioning in MDD patients with SA. Therefore, we speculate that the ReHo changes of ACC in depression and suicide should be similar, which may be related to the common pathophysiological mechanism of depression and suicide in this region.

Recent brain functional imaging studies have found that the precuneus is associated with many high levels cognitive functions, such as episodic memory, self-related information processing. Previous studies mostly focused on the local functional consistency of precuneus in depression, and little attention has been paid on suicide. Of the two suicide-related ReHo studies, one was that ReHo in the left precuneus was higher in the SA group than in the normal control group (Cao et al., 2015), the other study found lower ReHo of the right cuneus in the SA group compared with the NSA group (Chen et al., 2021). No such result was found in our study. However, there are many studies on ReHo indicators of depression. Liu et al. (2012) study found that compared to the healthy controls, MDD patients had significantly decreased ReHo in right precuneus. Moreover, other studies found that there were significant lower in ReHo in left anterior cingulate cortex and bilateral precuneus in MDD group compared with the control group (Lai, 2018). These are consistent with the results of our study. However, findings that are inconsistent with ours were reported, including those those in the study of Zhang et al. (2021) where it was shown that ReHo in the right precuneus lobe of patients with SA depression was significantly increased compared with healthy controls. Studies reported that relative to patients with LOD (later adult onset depression, age 30–44), patients with EOD (early adult onset depression, age 18–29) displayed significantly increased ReHo in the left precuneus (Chen et al., 2012; Shen et al., 2017). In our study, we did not have similar observations on the left precuneus, probably reflecting the idiosyncrasies of the different study subjects. The reason for the similarities and differences of these results may also be that precuneus is not necessarily a dysfunctional brain area caused by suicide, but may be a specific indicator of depression.

Regarding the correlation between ReHo and HAMD, a study found that the right cuneus of SI was positively correlated with HAM-D (Chen et al., 2021). Another study (Modinos et al., 2014) found that the volume of anterior cingulate gyrus negatively correlated with suicidal symptoms. The correlation analysis of another study (Zhang et al., 2021) showed that there was no significant correlation between the BDI-II score and the ReHo value of the precuneus in the SA group. Our study found that the mean ReHo in the right cingulate gyrus was correlated with HAMD-SA scores, but was not associated with HAMD-24 scores. It also indirectly illustrates that structural and functional abnormalities of the cingulate gyrus may be closely related to the symptoms of suicide.

Our study had some limitations: first, the sample size for MDD patients was relatively small. A larger sample size is needed to replicate the results we presented here. Second, our study was a cross-sectional design, and the data were insufficient to establish a causal relationship between depressive symptoms and suicidal behavior. Future studies using longitudinal designs will be useful for examining the causal relationship between depression and SA. Third, we only studied suicidal ideation in MDD patients. Future studies are needed to examine the role of the right cingulate gyrus in suicidal behavior in patients with other mental disorders, such as schizophrenia. Last but not least, it has been demonstrated that periods of untreated depression are associated with greater volume loss in some brain regions. We will certainly pay attention to this point in future studies. Therefore, our findings should be considered preliminary and should be confirmed before firm conclusions can be drawn.

## Conclusion

Our study showed that SA in depressed patients was associated with alterations in resting state brain activity. Our results suggest that the neural basis of psychopathology in depressed patients with suicidal ideation may involve functional abnormalities in multiple brain regions. The pattern of increased local functional activity in the right cingulate may be related to SA. Identifying cingulate activity may help elucidate the etiology and pathogenesis associated with SA.

## Data Availability Statement

The raw data supporting the conclusions of this article will be made available by the authors, without undue reservation.

## Ethics Statement

The studies involving human participants were reviewed and approved by Medical Research Ethics Committee of Shanxi Medical University. The patients/participants provided their written informed consent to participate in this study.

## Author Contributions

KZ and ZL designed and supervised this study. NS, YW, GL, and CY were responsible for data analysis and manuscript drafting. PL and LL revised the manuscript. YD and AZ participated in sample collection and carried out the experimental procedures. All authors reviewed and approved the final manuscript.

## Conflict of Interest

The authors declare that the research was conducted in the absence of any commercial or financial relationships that could be construed as a potential conflict of interest.

## Publisher’s Note

All claims expressed in this article are solely those of the authors and do not necessarily represent those of their affiliated organizations, or those of the publisher, the editors and the reviewers. Any product that may be evaluated in this article, or claim that may be made by its manufacturer, is not guaranteed or endorsed by the publisher.

## Footnotes

1
www.fil.ion.ucl.uk/spm
